# Pristine populations of habitat-forming gorgonian species on the Antarctic continental shelf

**DOI:** 10.1038/s41598-017-12427-y

**Published:** 2017-09-25

**Authors:** Stefano Ambroso, Janire Salazar, Rebeca Zapata-Guardiola, Luisa Federwisch, Claudio Richter, Josep Maria Gili, Nuria Teixidó

**Affiliations:** 1 Institut de Ciències del Mar (ICM-CSIC), Pg. Marítim de la Barceloneta 37-49, 08003 Barcelona, Spain; 2 Alfred-Wegener-Institut Helmholtz-Zentrum für Polar- und Meeresforschung, 27568 Bremerhaven, Germany; 3 University of Bremen, 28334 Bremen, Germany; 4 Stazione Zoologica Anton Dohrn, 80121 Naples, Italy; 5 Hopkins Marine Station, Stanford University, 120 Ocean view Blvd, Pacific Grove, CA 93950 USA

## Abstract

Declines in the abundance of long-lived and habitat-forming species on continental shelves have attracted particular attention given their importance to ecosystem structure and function of marine habitats. The study of undisturbed habitats defined as “pristine areas” is essential in creating a frame of reference for natural habitats free of human interference. Gorgonian species are one of the key structure-forming taxa in benthic communities on the Antarctic continental shelf. Current knowledge of the diversity, distribution and demography of this group is relatively limited in Antarctica. To overcome this lack of information we present original data on pristine and remote populations of gorgonians from the Weddell Sea, some of which display the largest colony sizes ever recorded in Antarctica. We assessed the distribution patterns of seven gorgonian species, a morphogroup and a family in front of the Filchner Ronne Ice Shelf (Weddell Sea) by means of quantitative analysis of video transects. Analysis of these videos showed a total of 3140 colonies of gorgonians with the highest abundance in the southern section and a significantly clumped distribution. This study contributes to the general knowledge of pristine areas of the continental shelf and identifies the eastern Weddell Sea as a hotspot for habitat-forming species.

## Introduction

The current state of the oceans is very different from what it was in the past^1,2^. Actually, most marine ecosystems are affected by climate change (*e.g*. ocean warming, acidification, sea level rise)^3–5^ and other multiple human-derived threats (*e.g*. overfishing, pollution, habitat destruction)^6–8^ which threaten marine global biodiversity and modify oceanic environments^5^ to the point of being considered “unnatural oceans”^9^ nearly devoid of “pristine” areas^10^. Such pristine areas are minimally affected by major human threats, thus providing a unique opportunity to better understand how marine ecosystems are structured and behave^11,12^. They are also essential to study the effects of climate change on benthic communities^13^, particularly on the Antarctic continental shelf where one can find still relatively undisturbed environments^14,15^. The potential impact of trawling activity has also become a major concern due to its extensive damage to continental shelves and deep cold-water coral reefs^16,17^. Although there is evidence of fishing activity as by-catch from longline fisheries in South Georgia^18^ and in the Ross Sea^19^, most of the Antarctic continental shelf has been little influenced by industrial fishing^20^. The lack of terrigenous sediments^21^, the relative constancy of its physical conditions^22,23^ and the relative absence of human-derived impacts^5^, make the Antarctic continental shelf a highly favourable environment for the development of high-density benthic megafauna communities.

In the last few decades, studies carried out on the continental shelf of the northeastern Weddell Sea have generated key insights on the diversity^24,25^, the degree of heterogeneity^26,27^, and the impact of iceberg scouring^28–30^. Iceberg scouring constitutes one of the major natural disturbances for high-Antarctic shelf fauna and it is increasingly apparent that iceberg scouring events may be altered by iceberg calving associated with regional atmospheric warming^31,32^. The estimated rate of disturbance of the Antarctic continental shelf by grounding icebergs is approximately 5%^26^, although still considerably less than the 53% attributable to trawling in other continental shelves^33^.

Gorgonians are among the main structural species of many benthic communities across all latitudes and depths, from shallow sublittoral habitats to continental shelves and deep seas^34–36^. Hence, the Commission for the Conservation of Antarctic Marine Living Resources (CCAMLR) has recognized gorgonians as a Vulnerable Marine Ecosystems (VME) indicator taxon^37^. These organisms contribute to the structure of benthic communities adding three-dimensional complexity to the habitat^38,39^. During the last decade, knowledge about diversity, distribution, ecology and state of conservation of gorgonian populations on the continental shelf has significantly increased in the Mediterranean Sea^40,41^, the Pacific Ocean^42,43^ and the Atlantic Ocean^44^. In Antarctica, most of the studies of this group of organisms have focused on taxonomy^45,46^, trophic ecology^47,48^, growth rates^49,50^ and reproductive ecology^51,52^. However, despite the high abundance of gorgonians in some locations of the Weddell Sea and their ecological role in Antarctic benthic communities^30,53^, there is still an important lack of knowledge on their ecological characteristics such as spatial distribution, abundance and demographic processes^54^.

Non-destructive sampling techniques like video-equipped towed gear, Remotely Operated Vehicles (ROVs) or manned submersibles are commonly used to study coastal areas^55^, deep reefs of cold-water corals^56^, seamounts^57^, and mesophotic areas^58^ to assess biodiversity patterns, characterize communities, evaluate spatial and temporal changes, and assess benthic ecosystem health status^59,60^. Although the majority of studies on Antarctic benthos have been carried out using semi-quantitative techniques like Agassiz and bottom trawls^61,62^, non-destructive image methodology has also been commonly used in the high Antarctic to provide quantitative information on the distributional patterns of benthic megafauna communities over large spatial and bathymetrical domains^28,53,58,63–65^.

Knowledge of demographic processes and spatial distribution patterns is a prerequisite to understand their role in benthic communities and provide basic information on their underlying dynamics and resilience, as well as to facilitate their management and conservation^66,67^. The major aim of the present study was to assess the health status of Antarctic gorgonian assemblages in a pristine and remote area in the southernmost part of the Weddell Sea continental shelf. Specifically, (1) we characterized the diversity and the abundance of gorgonians group; (2) explored their distribution patterns, and (3) assessed their population size structure. This study attempts to be a benchmark for the investigation of continental shelf habitats modified by anthropogenic pressure and to contribute to the general knowledge of pristine areas with habitat-forming species.

## Results

### Abundance of gorgonian populations

**Figure 1:**
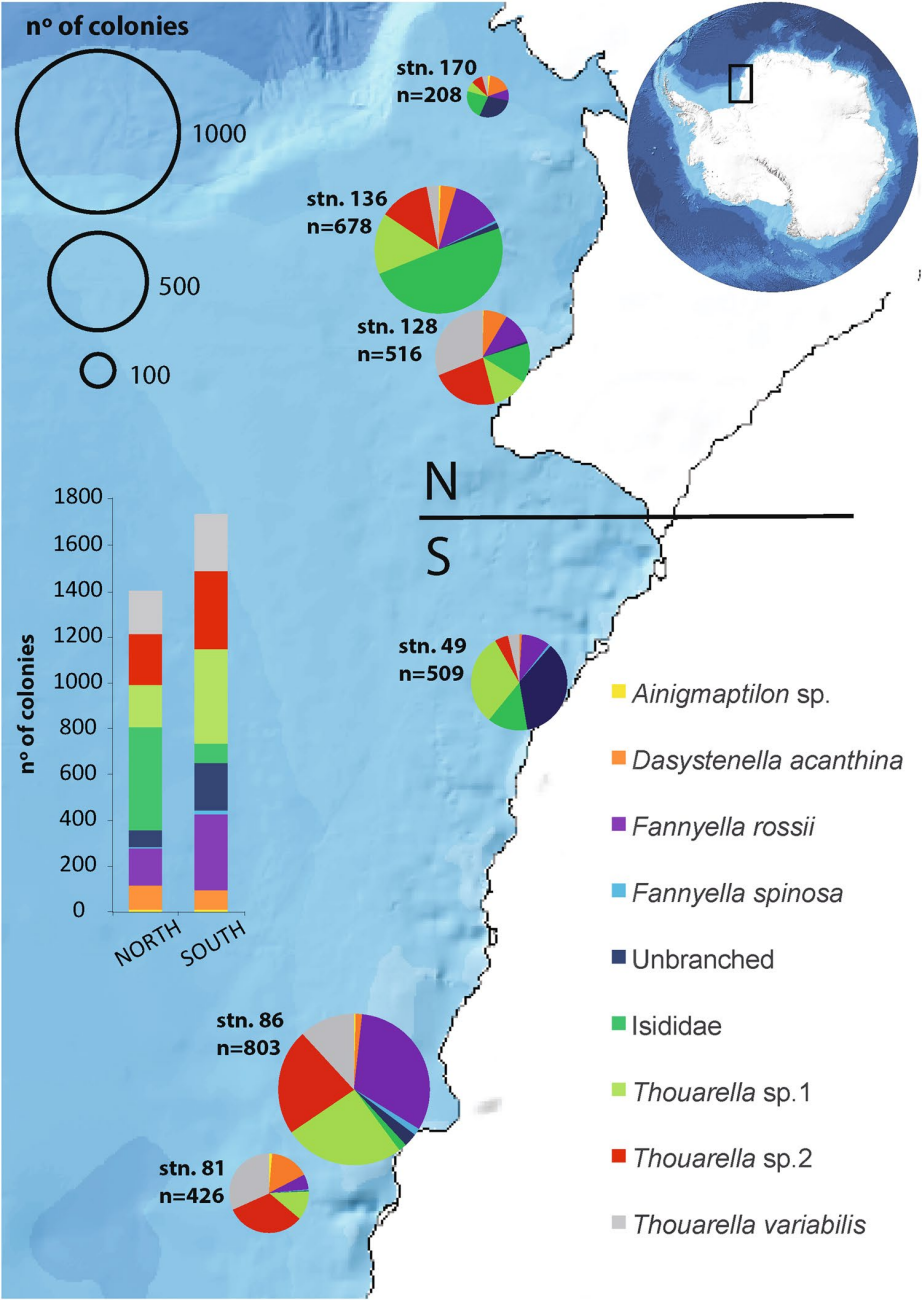
Composition, distribution and abundance of gorgonian species. The pie charts display the percentage of gorgonian taxa at each transect (n = number of colonies per transect). The size of the pie charts represents the abundance of the gorgonians. The histogram shows the abundance of gorgonian species per region (north: stations 128, 136, 170; south 49, 81, 86). Map of Antarctica was downloaded from http://www.ibcso.org/data.html ^100^. The figure was generated with QGIS Version 2.12 http://www.qgis.org/it/site/.

A total of 3140 colonies (1402 in the north and 1738 in the south), comprising seven gorgonian species (*Ainigmaptilon* sp., *Dasystenella acanthina*, *Fannyella rossii*, *Fannyella spinosa, Thouarella* sp.1, *Thouarella* sp.2, and *Thouarella variabilis*), an unbranched morphogroup (which included specimens from the genus *Onogorgia*, *Armadillogorgia*, *Primnoella* and *Arntzia*) and the family Isididae, were counted along six transects (Supplementary Table S1), occurring with a frequency of 64.4% in 1836 sampling units of 1 m^2^. Based on our count data, we estimated more than 46000 and more than 97000 colonies for the north and the south region, respectively. Overall, *Thouarella* sp.1 was the most abundant (n = 597 total colonies across all transects) and the second most frequent species representing 19% of observed colonies present in 20% of the sampling units. *Thouarella* sp.2 was the second most abundant (n = 572), but the most frequent (20%) species. The family Isididae (n = 535, 17.8%), *Fannyella rossii* (n = 474, 17.5%) and *Thouarella variabilis* (n = 438, 15.4%) were the third, fourth and fifth most abundant and frequent species, respectively. The unbranched group (n = 280) was more abundant than *Dasystenella acanthina* (n = 189), but less frequent (5.4% and 7.5%, respectively). The other species accounted for less than 2% of the observed colonies, occurring in less than 3% of the sampling units. Generally, abundance of the gorgonians differed between the two study sections, being lower in the northern part. Only Isididae (n = 277) and *Dasystenella acanthina* (n = 87) abundance showed high values in the northern stations (Fig. 1).

### Spatial distribution

**Figure 2:**
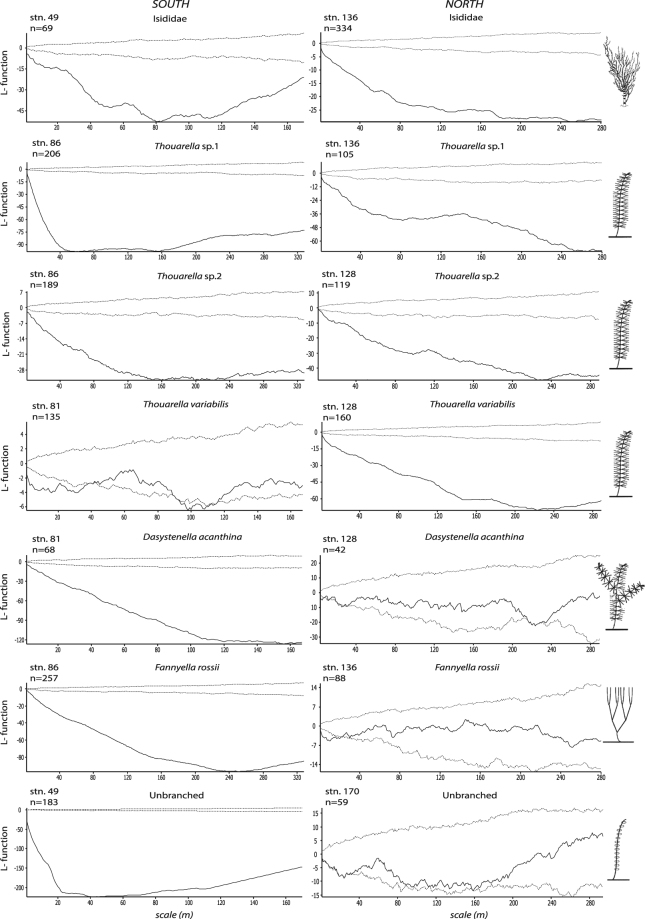
L-function (Ripley’s K) for the most abundant species. Values below 95% confidence interval (dotted lines) indicate a statistically significant clumped distribution of colonies; values within the confidence interval indicate a random distribution; values above the confidence interval indicate a statistically significant over-dispersed distribution (n = number of colonies).

**Figure 3:**
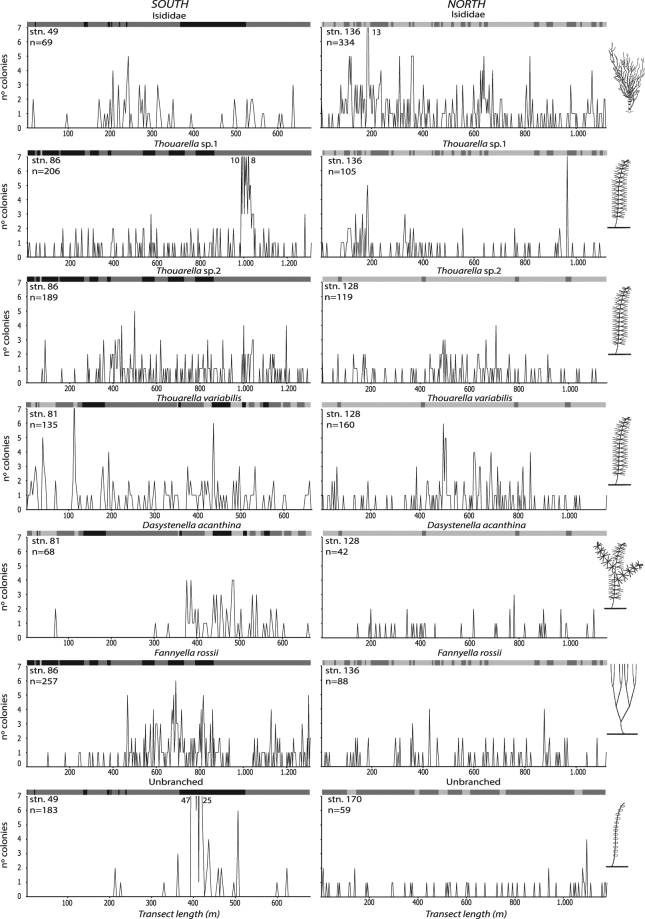
Density plots. Densities of the most abundant species are plotted at each station (n = number of colonies). Substrate type is indicated as black (coarse sediment), dark grey (intermediate sediment) and light grey (fine sediment).

Ripley’s K analysis revealed a significantly clumped distribution of the family Isididae, *Thouarella* sp.1 and *Thouarella* sp.2 colonies at all scales (from 1 m^2^ to whole transect). *Thouarella variabilis* showed a clumped distribution in the north, but a random distribution in the south. An opposite result for spatial distribution was found for *Dasystenella acanthina, Fannyella rossii* and Unbranched (Fig. 2). Gorgonian abundances varied markedly among the various stations (F_5,1179_ = 53.3, *p* < 0.001) (Fig. 3). In stn. 49, the Unbranched morphogroup was the group of gorgonians with the highest abundance (47 col/m^2^) and mean density of 7.3 ± 11.5 col/m^2^, while the least abundant species were *Fannyella spinosa* and *Ainigmaptillon* sp., with highest abundance of 20 and 19 col/m^2^ and mean density of 1.3 ± 0.6 and 1.2 ± 0.4 col/m^2^, respectively (Fig. 3).

### Population size structure

**Figure 4:**
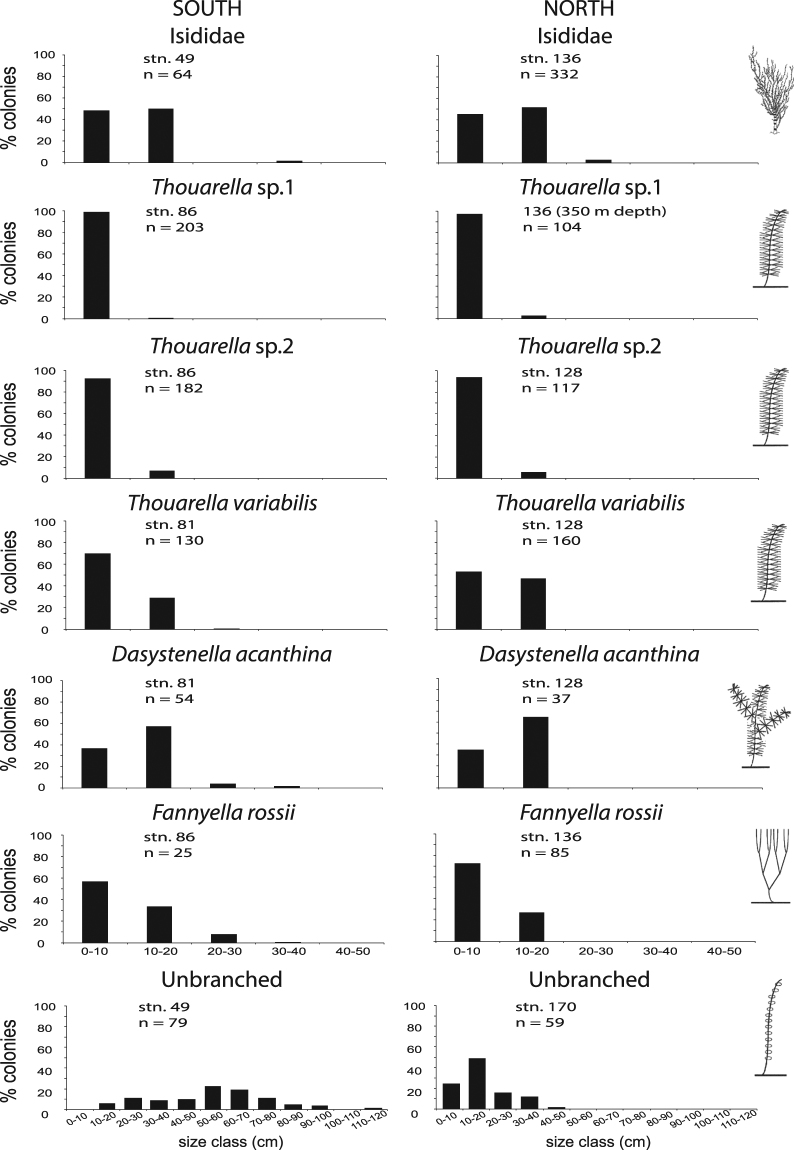
Size-frequency distribution of gorgonian populations (n = number of colonies).

The size-frequency distributions of the 7 species populations were unimodal (Fig. 4). Most of the gorgonian populations were positively skewed, indicating an asymmetrical distribution of size frequency and a high proportion of small colonies (0–10 cm) (Fig. 4 and Supplementary Table S2). In contrast, the two populations of the Unbranched morphogroup were not skewed, being dominated by medium-sized colonies (10–20 cm in the south and 50–60 cm in the north) (Fig. 4 and Supplementary Table S2). Most of the northern populations showed no significant kurtosis while in the south all the gorgonian populations, except Unbranched, showed significant kurtosis (Supplementary Table S2). Finally, in both studied areas, all gorgonian populations displayed the same size class distributions (Fig. 4).

### Population structure

**Figure 5:**
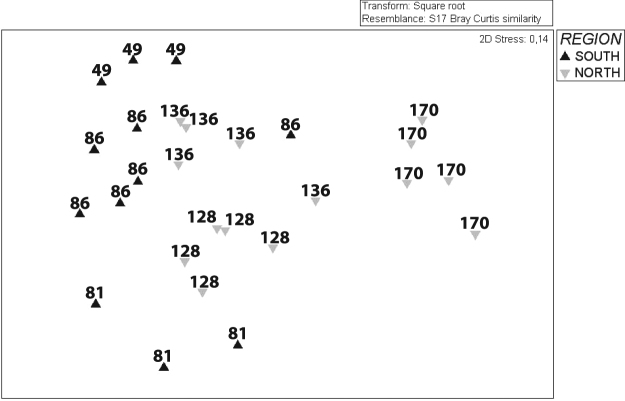
Non-metric multidimensional scaling (nMDS) ordination plot of abundance of gorgonian species in the south and north region of the SE Weddell Sea. Analysis performed on Bray-Curtis dissimilarities for abundance (colonies/m^2^). Each point represents a subsample of 200 m length for each station.

The structure of gorgonian populations gradually differed in both areas, with significant differences between north and south (F_1,1183_ = 65.06, *p* < 0.001) (Fig. 5).

The SIMPER analysis showed an average similarity in species composition which ranged from 17.89% to 31.68% (Supplementary Table S3). The number of species contributing up to 90% of the similarity was the same in the two sections (Supplementary Table S3). *Thouarella* sp.1 contributed most (25.14%) to the similarity in the northern transects, while the family Isididae was especially relevant in the southern transects (22.43% of contribution). Focusing on taxa dissimilarities, the family Isididae was the most important contributing with 17% of the average dissimilarity between north and south.

## Discussion

This study focused on previously unknown extensive gorgonian assemblages in the southeastern Weddell Sea. Our results indicate that this region is a hotspot for gorgonian diversity in terms of both number of species and their abundance. The diversity of the studied gorgonian assemblages was similar to those reported on subtropical^68^, Mediterranean shelf^41^ and other Antarctic coastal areas^69^. Seven different species, a gorgonian morphogroup and a gorgonian family Isididae were observed to dwell between 250 and 350 m depth within the study area (Fig. 1), in agreement with previous findings in coastal areas of the Antarctic Peninsula^69^. On the other hand, gorgonian density observed in these multi-specific assemblages clearly exceeds (by ten-fold; 47 colonies/m^2^) those reported for other Antarctic and Arctic shelf areas^70,71^ (Supplementary Table S4). These high density values were similar to those found in temperate^72,73^ and tropical^74,75^ coastal assemblages (Supplementary Table S4). Despite extreme environmental conditions and the general theory that species richness decreases with increasing latitude, it is also generally accepted that this theory is not strictly true and varies with species in the Southern Ocean^76,77^. In addition, benthic biomass in some Antarctic areas is larger than in temperate and subtropical areas^78^. Such dense three-dimensional communities cover large sections of the Antarctic continental shelf as well as the Mediterranean Sea^41^ and deep undisturbed North Atlantic coral banks^44^. *Fannyella rossii* and the three species of genus *Thouarella* showed high abundances in each video transect (Supplementary Table S1). This highlights the unique abundance of these Antarctic gorgonian species^45^. Of additional note is their high density, with maximum values of 6 ind/m^2^ for *Fannyella rossii* and 10 ind/m^2^ for *Thouarella* sp.1 in the southern section (Supplementary Table S1).

All populations in this study were represented by many small colonies with a positively skewed colony size distribution (Fig. 4). The size structure of a population results from the action of biotic and abiotic factors and from the type, intensity, and frequency of disturbance to which individuals are exposed^79,80^. Positively skewed size frequency distribution implies that a population is in a healthy state and growing, since it includes an abundance of juveniles^81,82^. On the contrary, a negative skewness indicates a lack of recent recruitment and therefore it implies a risk of population decline^81,83^. Population size structure of all *Thouarella* and *Fannyella* species was mostly asymmetrical with many small colonies (Fig. 4), suggesting high recruitment rates^82^. The size structure also reflects the growth and the development of each individual within the population, as well as past recruitment and mass mortality events. Unfortunately, due to their inaccessibility, only a few studies of gorgonian population size structure have been done on continental shelves^41^. Unbranched individuals seem to grow slowly and older without being replaced (low recruitment) with few small colonies (sexual juveniles) and dominance of large-sized individuals^41^.

As a rule of thumb, because of their slow growth rate^84,85^ and reproduction type, gorgonians are especially vulnerable to iceberg scouring^86,87^, making their recovery very slow^17^. All gorgonian species described in this study reproduce by internal brooding. This means that the settlement of the larvae occurs at short distances from the parents^88^ explaining the patchy distribution along all video transects. Some gorgonian species such as *Ainigmaptillon antarcticum* and *Primnoisis antarctica*, which are internal brooders, are also pioneer taxa appearing during the first stage of recolonization after iceberg scouring events with patchy distribution^86,87^.

The high diversity and abundance of gorgonian assemblages on the Antarctic continental shelf, and the vast area covered by high densities of genus *Thouarella* are probably related to the low iceberg scouring pressure and oceanographic-ice conditions. Constant hydrodynamic conditions that increase particle suspension in the near-bottom water layers may also imply enhanced food availability for gorgonians on the continental shelf^89^. Strong currents are advantageous to the establishment of this group of organisms supplying them with food and continuously keeping them completely clear of sediment^90,91^. Moreover, based on our findings of high abundance and large sizes in the southern section of our study area, we hypothesize that it is little affected by iceberg scouring, thereby favouring the establishment of well-developed pristine gorgonian populations.

Reduced abundance of long-lived and habitat-forming species from the deep sea and continental shelves in shallow sublittoral habitats have attracted particular attention, given their disproportionate importance to ecosystem structure and function, and the social value of marine habitats^14^. Yet, factors responsible for such decline are mainly overexploitation and habitat destruction by bottom trawling and by-catch fishing^92^. Evidence of fishing activities with *Thouarella* spp. as by-catch has been reported in South Georgia^18^. Moreover, specimens from the genus *Primnoa* and the family Isididae from longline fisheries were found in the Ross Sea^19^. To our knowledge, our results are the first to show pristine populations of gorgonians with the highest abundance and largest size ever recorded on the Antarctic continental shelf. These populations are far more mature and better preserved than any other known population in Antarctica. Clearly, more research is needed to determine the locations of such refuges and to devise strategies to protect such gorgonian populations as well as the many other species interacting with them. The study of these pristine gorgonian populations may also provide basic knowledge on how other continental shelf and upper slope communities may have thrived in the decades before bottom trawling fishing ensued.

## Methods

### Study area

The study area was sampled as part of the multidisciplinary PS82 (ANT XXIX/9) expedition on board R/V *Polarstern* from December 19, 2013, to March 5, 2014^93^. It is located in front of the Filchner Ronne Ice Shelf in the southernmost part of the Weddell Sea; a region poorly investigated due to the heavy sea ice conditions^93^. The small amount of data available from this area has made it an area of special relevance to better understand oceanographic conditions and to gain new insights into biodiversity patterns in this remote and pristine region^94^. The study area was divided into a south and a north section due to Brunt Ice Shelf, which may produce different oceanographic conditions^93^.

### ROV sampling procedure

In order to study the composition and distribution of gorgonians, an inspection-class ROV (Remotely Operated Vehicle, Ocean Modules V8 Sii) was deployed at six stations in the area of the Filchner Trough (Supplementary Table S5). Three random stations (stn. 49, stn. 81, stn. 86) were recorded in the southern part of the continental shelf and three random stations (stn. 128, stn. 136, stn. 170) in its northern part closer to the shelf break. The ROV was equipped with a High Definition (HD) video camera (Kongsberg oe14–502) looking forward in an angle of 40–45° and two parallel lasers providing a reference scale of 4 cm on the video (see Knust and Schröder 2014^93^ for more details on the ROV procedure). The ROV video material is available from the data publisher PANGEA at www.pangea.de (see Table S5 for DOIs).

### Species identification

In order to confirm the taxonomic identification of the species observed in the videos, colonies of gorgonians were collected with an Agassiz Trawl (AGT) after the ROV deployments. Colonies were fixed and preserved in 10% formalin until analysed in the laboratory (see Supplementary Table S6 for taxonomic remarks on the identification of some groups of species). We identified 7 gorgonian species belonging to the family *Primnoidae* (*Ainigmaptilon* sp., *Dasystenella acanthina*, *Fannyella rossii*, *Fannyella spinosa, Thouarella* sp.1, *Thouarella* sp.2, *and Thouarella variabilis*), an unbranched morphogroup (flagelliform colonies with polyps distributed in whorls along the main stem of the colony), which included specimens of the genera *Onogorgia*, *Armadillogorgia*, *Primnoella* and *Arntzia*, and a bamboo coral group of the Family Isididae, (Supplementary Figures S1 and S2).

### Video analysis

Quantitative video analysis was performed using the software SONY XDCAM Viewer. Every gorgonian observed within a width of 0.3 m (based on the laser beams) along each video transect was identified with a distance from the beginning of the transect according to the ROV’s ultra-short baseline (USBL) position data.

### Spatial distribution and size structure

We examined the species composition and quantified the frequency as the relative proportion of each species present for each sampling unit of the transect and the abundance as the total number of colonies across all the transects (see below). The most abundant species of gorgonians were used to compare their abundance, spatial distribution and size class in both the north and south areas. These results were displayed in density plots, obtained by transforming each transect into a string of contiguous quadrats of 1 m^2^ (0.3 × 3.33 m) and counting the number of colonies of each species only inside each quadrat. A total of 1836 useful sampling units were obtained from the 6 transects.

The significance of the deviation from a random distribution was analysed with the one-dimensional version of Ripley’s K-function second-order spatial statistic^95,96^. When the sample statistic is found within the bounds of the confidence interval at any point, it indicates complete spatial randomness; a significant positive deviation of the sample statistic indicates over-dispersion of the colonies, whereas a significant negative deviation indicates a clumped distribution^67^.

To study population size structure, the maximum height of each observed gorgonian colony was measured using the Macnification 2.0.1 software on still images extracted from recorded footage^97^. The distance between the two laser beams was used to calibrate extracted images and measurements were performed on still images in which the laser beams were in the same plane as the colony base to reduce the error due to the perspective^55^. Based on previous studies, colony size class was defined for each 10 cm^55,82^. We considered as young colonies the smallest colonies that could be distinguished using the video analysis (2–5 cm in height)^80^. Size structure was also analysed in terms of descriptive statistics using distribution parameters such as skewness and kurtosis. Skewness is a measure of the symmetry of a distribution using its mean, reflecting the proportion of small versus large colonies in a gorgonian population; if skewness is significant the distribution is asymmetric. Kurtosis is a measure of the peakedness of a distribution near its central mode. A significant kurtosis value indicates longer tails than would be expected for a normal distribution, and therefore a particular colony size prevails in the population. Only transects with more than 40 colonies were studied for population size structure in order to generate meaningful skewness and kurtosis estimates.

### Population size structure and density data from other areas of the continental shelf

To compare our data with that of other gorgonian populations dwelling on the continental shelf of other seas, we compiled data on maximum abundance, mean density and maximum height from previous studies using ROV observations. Overall, we compiled population structure data for 36 taxa and 12 different study areas (Supplementary Table S4).

### Assemblage structure

A non-metric multi-dimensional scaling (nMDS) ordination analysis was performed based on the Bray–Curtis similarity measure using square-root-transformed abundance data. For visualization purposes, data are presented for each 200 m length. Furthermore, a similarity percentage procedure analysis, SIMPER^98^, was performed to identify the relative contribution of each species to average similarities between areas. A non-parametric analysis of variance, PERMANOVA^99^, was applied using Bray-Curtis distance for the multivariate analyses. Statistical analyses were computed using the program Primer v6 with the PERMANOVA + add-on package.

## Electronic supplementary material

Supplementary Information
