# Supplementary material for: Pristine populations of habitat-forming gorgonian species on the Antarctic continental shelf
Source: Sci Rep. 2017 Sep 25;7:12251. doi: 10.1038/s41598-017-12427-y (PMC5612998; doi:10.1038/s41598-017-12427-y)
Supplement: Supplementary file 1 — Supplementary Information [file 41598_2017_12427_MOESM1_ESM.doc]

Title

**Pristine populations of habitat-forming gorgonian species on the Antarctic continental shelf**

Stefano Ambroso, Janire Salazar, Rebeca Zapata-Guardiola, Luisa Federwisch, Claudio Richter, Josep Maria Gili, Nuria Teixidó.

Supplementary Table S1 – Gorgonian presence and spatial distribution in the northern subarea. Occurrence (frequency of occurrence in the set of sampling units) is given for each transect and species; abundance (number of colonies) and maximum density of each species is given for each transect.

Supplementary Table S2 – Size structure characteristics of the studied gorgonians species: colony height, skewness and kurtosis. Asterisks indicate the degree of significance.

Supplementary Table S3 – SIMPER analysis: (a) Species that cumulatively contribute to 90% of the average similarities within the regions (Aver. Sim), (b) Species that contribute cumulatively to 90% of the average dissimilarities between the regions (Aver. Diss).

Supplementary Table S4 – Summary of gorgonian data reported from previous ROV observations from different continental shelfs. Data shows maximum abundance, mean density and maximum height.

Supplementary Table S5 – ROV deployments during PS82 (ANT XXIX/9).

Supplementary Table S6 – Remarks on the identification of gorgonian species.

Supplementary Figure S1 – Images of studied species collected with Agassiz trawl. a) Isididae, b) *Thouarella variabilis*, c) *Thouarella* sp.1, d) *Thouarella* sp.2, e) *Dasystenella acanthina*, f) *Fannyella rossii*, g) *Fannyella spinosa*, h) Unbranched, i) *Ainigmaptilon* sp. Scale Bar: 4 cm.

Supplementary Figure S2 – Studied species images from ROV videos. a) Isididae, b) *Thouarella variabilis*, c) *Thouarella* sp.1, d) *Thouarella* sp.2, e) *Dasystenella acanthina*, f) *Fannyella rossii*, g) *Fannyella spinosa*, h) Unbranched, i) *Ainigmaptilon* sp. Scale Bar: 4 cm. All photos taken from video material. © Claudio Richter, AWI, Germany.

Supplementary Table S1

| Station | Sampling Units | | | | | *Species* | | Sampling units per species | | | | Colonies | | | | Mean density ± SD | | | Max density | |
| --- | --- | --- | --- | --- | --- | --- | --- | --- | --- | --- | --- | --- | --- | --- | --- | --- | --- | --- | --- | --- |
|  | Number | with gorgonians | | (%) | |  |  | Number | | (%) | | Number | | (%) | | (colonies · m^-2^) | | (colonies · m^-2^) | | |
| 128 | 348 | 234 | | (67.2) | | *Ainigmaptilon* sp. | | 2 | | (0.6) | | 2 | | (0.4) | | 1 ± 0 | | 1 | | |
|  |  |  |  |  |  | ***Dasystenella acanthina*** | | **33** | | **(9.5)** | | **42** | | **(8.1)** | | **1.3 ± 0.5** | | **3** | | |
|  |  |  |  |  |  | *Fannyella rossii* | | 50 | | (14.4) | | 56 | | (10.9) | | 1.1 ± 0.4 | | 3 | | |
|  |  |  |  |  |  | *Thouarella* sp.1 | | 50 | | (14.4) | | 63 | | (12.2) | | 1.3 ± 0.5 | | 3 | | |
|  |  |  |  |  |  | ***Thouarella* sp.2** | | **91** | | **(26.1)** | | **119** | | **(23.1)** | | **1.3 ± 0.6** | | **4** | | |
|  |  |  |  |  |  | ***Thouarella variabilis*** | | **102** | | **(29.3)** | | **160** | | **(31.0)** | | **1.3 ± 1.0** | | **6** | | |
|  |  |  |  |  |  | Unbranched | | 4 | | (1.1) | | 4 | | (0.8) | | 1 ± 0 | | 1 | | |
|  |  |  |  |  |  | Isididae | | 54 | | (15.5) | | 70 | | (13.6) | | 1.3 ± 0.6 | | 4 | | |
| 136 | 336 | 249 | | (74.1) | | *Ainigmaptilon* sp. | | 4 | | (1.2) | | 4 | | (0.6) | | 1 ± 0 | | 1 | | |
|  |  |  |  |  |  | *Dasystenella acanthina* | | 23 | | (6.8) | | 27 | | (4.0) | | 1.2 ± 0.4 | | 2 | | |
|  |  |  |  |  |  | ***Fannyella rossii*** | | **62** | | **(18.5)** | | **88** | | **(13.0)** | | **1.4 ± 0.7** | | **4** | | |
|  |  |  |  |  |  | *Fannyella spinosa* | | 3 | | (0.9) | | 4 | | (0.6) | | 1.3 ± 0.6 | | 2 | | |
|  |  |  |  |  |  | ***Thouarella* sp.1** | | **74** | | **(22.0)** | | **105** | | **(15.5)** | | **1.4 ± 1.0** | | **7** | | |
|  |  |  |  |  |  | *Thouarella* sp.2 | | 65 | | (19.3) | | 86 | | (12.7) | | 1.3 ± 0.6 | | 4 | | |
|  |  |  |  |  |  | *Thouarella variabilis* | | 14 | | (4.2) | | 20 | | (2.9) | | 1.4 ± 0.7 | | 3 | | |
|  |  |  |  |  |  | Unbranched | | 10 | | (3.0) | | 10 | | (1.5) | | 1 ± 0 | | 1 | | |
|  |  |  |  |  |  | **Isididae** | | **181** | | **(53.9)** | | **334** | | **(49.3)** | | **1.8 ± 1.5** | | **13** | | |
| 170 | 354 | 147 | | (41.5) | | *Ainigmaptilon* sp*.* | | 4 | | (1.1) | | 4 | | (1.9) | | 1 ± 0 | | 1 | | |
|  |  |  |  |  |  | *Dasystenella acanthina* | | 31 | | (8.8) | | 37 | | (17.8) | | 1.2 ± 0.5 | | 3 | | |
|  |  |  |  |  |  | *Fannyella rossii* | | 17 | | (4.8) | | 18 | | (8.7) | | 1.1 ± 0.2 | | 2 | | |
|  |  |  |  |  |  | *Thouarella* sp.1 | | 15 | | (4.2) | | 16 | | (7.7) | | 1.1 ± 0.3 | | 2 | | |
|  |  |  |  |  |  | *Thouarella* sp.2 | | 18 | | (5.1) | | 18 | | (8.7) | | 1 ± 0 | | 1 | | |
|  |  |  |  |  |  | *Thouarella variabilis* | | 9 | | (2.5) | | 9 | | (4.3) | | 1 ± 0 | | 1 | | |
|  |  |  |  |  |  | **Unbranched** | | **52** | | **(14.7)** | | **59** | | **(28.4)** | | **1.1 ± 0.5** | | **4** | | |
|  |  |  |  |  |  | Isididae | | 42 | | (11.9) | | 47 | | (22.6) | | 1.1 ± 0.3 | | 2 | | |
| 49 | 204 | 138 | (67.7) | | *Ainigmaptilon* sp. | | 1 | | (0.5) | | 1 | | (0.2) | | 1 ± 0 | | 1 | | |  |
|  |  |  |  |  | *Dasystenella acanthina* | | 4 | | (2.0) | | 4 | | (0.8) | | 1 ± 0 | | 1 | | |  |
|  |  |  |  |  | *Fannyella rossii* | | 30 | | (14.7) | | 49 | | (9.6) | | 1.6 ± 0.9 | | 4 | | |  |
|  |  |  |  |  | *Fannyella spinosa* | | 3 | | (1.5) | | 4 | | (0.8) | | 1.3 ± 0.6 | | 2 | | |  |
|  |  |  |  |  | *Thouarella* sp.1 | | 88 | | (43.1) | | 157 | | (30.8) | | 1.8 ± 1.3 | | 7 | | |  |
|  |  |  |  |  | *Thouarella* sp.2 | | 18 | | (8.8) | | 23 | | (4.5) | | 1.3 ± 0.5 | | 2 | | |  |
|  |  |  |  |  | *Thouarella variabilis* | | 7 | | (3.4) | | 19 | | (3.7) | | 2.7 ± 1.8 | | 5 | | |  |
|  |  |  |  |  | **Unbranched** | | **25** | | **(12.3)** | | **183** | | **(36.0)** | | **7.3 ± 11.5** | | **47** | | |  |
|  |  |  |  |  | **Isididae** | | **39** | | **(19.1)** | | **69** | | **(13.6)** | | **1.8 ± 1** | | **5** | | |  |
| 81 | 200 | 139 | (69.5) | | *Ainigmaptilon* sp. | | 5 | | (2.5) | | 6 | | (1.4) | | 1.2 ± 0.4 | | 2 | | |  |
|  |  |  |  |  | ***Dasystenella acanthina*** | | **37** | | **(18.5)** | | **68** | | **(16.0)** | | **1.8 ± 1** | | **4** | | |  |
|  |  |  |  |  | *Fannyella rossii* | | 15 | | (7.5) | | 26 | | (6.1) | | 1.7 ± 1.1 | | 4 | | |  |
|  |  |  |  |  | *Fannyella spinosa* | | 2 | | (1.0) | | 2 | | (0.5) | | 1 ± 0 | | 1 | | |  |
|  |  |  |  |  | *Thouarella* sp.1 | | 35 | | (17.5) | | 50 | | (11.7) | | 1.4 ± 0.7 | | 4 | | |  |
|  |  |  |  |  | *Thouarella* sp.2 | | 72 | | (36.0) | | 137 | | (32.2) | | 1.9 ± 1.1 | | 5 | | |  |
|  |  |  |  |  | ***Thouarella variabilis*** | | **81** | | **(40.5)** | | **135** | | **(31.7)** | | **1.7 ± 1.2** | | **8** | | |  |
|  |  |  |  |  | Unbranched | | 1 | | (0.5) | | 1 | | (0.2) | | 1 ± 0 | | 1 | | |  |
|  |  |  |  |  | Isididae | | 1 | | (0.5) | | 1 | | (0.2) | | 1 ± 0 | | 1 | | |  |
| 86 | 394 | 276 | (70.1) | | *Ainigmaptilon* sp. | | 3 | | (0.8) | | 3 | | (0.4) | | 1 ± 0 | | 1 | | |  |
|  |  |  |  |  | *Dasystenella acanthina* | | 11 | | (2.8) | | 11 | | (1.4) | | 1 ± 0 | | 1 | | |  |
|  |  |  |  |  | ***Fannyella rossii*** | | **150** | | **(38.1)** | | **257** | | **(31.7)** | | **1.7 ± 1** | | **6** | | |  |
|  |  |  |  |  | *Fannyella spinosa* | | 12 | | (3.0) | | 12 | | (1.5) | | 1 ± 0 | | 1 | | |  |
|  |  |  |  |  | ***Thouarella* sp.1** | | **107** | | **(27.2)** | | **206** | | **(25.4)** | | **1.9 ± 1.7** | | **10** | | |  |
|  |  |  |  |  | ***Thouarella* sp.2** | | **126** | | **(32.0)** | | **189** | | **(23.3)** | | **1.5 ± 0.8** | | **5** | | |  |
|  |  |  |  |  | *Thouarella variabilis* | | 70 | | (17.8) | | 95 | | (11.7) | | 1.4 ± 0.9 | | 7 | | |  |
|  |  |  |  |  | Unbranched | | 17 | | (4.3) | | 23 | | (2.8) | | 1.4 ± 1.2 | | 6 | | |  |
|  |  |  |  |  | Isididae | | 11 | | (2.8) | | 14 | | (1.7) | | 1.3 ± 0.5 | | 2 | | |  |

Supplementary Table S2

Supplementary Table S3

| **a) Similarity** |  |  |  |
| --- | --- | --- | --- |
| **Species** | **Sim /SD** | **Contribution%** | **Cum. Contribution%** |
| **Group South (Aver. Sim=65%)** |  |  |  |
| *Thouarella* sp.1 | 3.7 | 25.1 | 25.1 |
| *Thouarella* sp.2 | 2.6 | 22.6 | 47.7 |
| *Fannyella rossii* | 2.8 | 18 | 65.8 |
| *Thouarella variabilis* | 1,9 | 16.6 | 82.4 |
| Isididae | 0.9 | 4.8 | 87.2 |
| *Dasystenella acanthina* | 0.9 | 4.7 | 91.9 |
| **Group North (Aver. Sim=65%)** | |  |  |
| Isididae | 2.4 | 22.4 | 22.4 |
| *Thouarella* sp.2 | 2.1 | 17 | 39.5 |
| *Dasystenella acanthina* | 2.9 | 15.6 | 55.1 |
| *Fannyella rossii* | 2.7 | 15.1 | 70.2 |
| *Thouarella* sp.1 | 1.8 | 13.3 | 83.5 |
| *Thouarella variabilis* | 1.1 | 9.9 | 93.4 |
| **b) Dissimilarity** |  |  |  |
| **Species** | **Diss /SD** | **Contribution%** | **Cum. Contribution%** |
| **Groups South & North**  **(Aver. Diss=43%)** |  |  |  |
| Isididae | 1.4 | 16.6 | 16.6 |
| *Thouarella* sp.1 | 1.2 | 15.2 | 31.8 |
| *Thouarella variabilis* | 1.4 | 13.8 | 45.7 |
| *Thouarella* sp.2 | 1.3 | 12.6 | 58.3 |
| *Fannyella rossii* | 1.1 | 11.9 | 70.2 |
| Unbranched | 0.7 | 10.8 | 81 |
| *Dasystenella acanthine* | 1.5 | 9 | 90 |
| *Fannyella spinosa* | 1.1 | 6.6 | 96.5 |

Supplementary Table S4

| Species | Max density (ind./m^2^) | Mean density (ind./m^2^) | Maximum size (cm) | Study area | Depth (m) | Reference |
| --- | --- | --- | --- | --- | --- | --- |
| *Acanella arbuscula* | 4.7 |  |  | Canada (Gully Canyon) | 404-540 | Mortensen and Buhl-Mortensen 2005 |
| *Acanthogorgia armata* | 1.99 |  |  | Canada (Gully Canyon) | 231-364 | Mortensen and Buhl-Mortensen 2004 |
| *Acanthogorgia armata* | 0.5 |  |  | Canada (Gully Canyon) | 346-493 | Mortensen and Buhl-Mortensen 2005 |
| *Acanthogorgia hirsuta* |  | 0.04 ± 0.2 |  | Tirrenean Sea | 200 - 250 | Bo et al. 2013 |
| *Acanthogorgia hirsuta* | 5 | 1.0 ± 1.0 |  | Western Mediterranean (Menorca channel) | 100 - 180 | Grinyó et al. 2016 |
| *Bebrice mollis* | 3 | 1.2 ± 0.3 |  | Western Mediterranean (Menorca channel) | 180 - 340 | Grinyó et al. 2016 |
| *Callogorgia verticillata* | 2 | 0.4 ± 0.07 |  | Tirrenian Sea | 200 - 250 | Bo et al. 2013 |
| *Callogorgia verticillata* | 5 | 1.0 ± 0.9 | 115.3 | Western Mediterranean (Menorca channel) | 100 - 180 | Grinyó et al. 2016 |
| *Corallium lauuense* |  | 0.33 ± 0.63 | 20.3 | Hawaii | 350 - 500 | Parrish 2007 |
| *Corallium secundum* |  | 0.56 ± 0.65 | 17.9 | Hawaii | 350 - 500 | Parrish 2007 |
| *Eunicella cavolonii* | 24 | 2.9 ± 2.7 | 50.4 | Western Mediterranean (Menorca channel) | 100 - 180 | Grinyó et al. 2016 |
| *Keratoisis ornata* | 0.54 |  |  | Canada (Gully Canyon) | 396-509 | Mortensen and Buhl-Mortensen 2005 |
| *Keratoisisi* sp. |  | 0.05 ± 0.1 |  | Bering Sea | 466 - 533 | Miller et al. 2012 |
| *Paragorgia arborea* | 0.49 |  |  | Canada (Gully Canyon) | >235 | Mortensen and Buhl-Mortensen 2004 |
| *Paragorgia arborea* | 0.6 |  | 180 | Canada (Gully Canyon) | 341 - 495 | Mortensen and Buhl-Mortensen 2005 |
| *Paramuricea macrospina* | 9 | 1.5 ± 1.7 | 55.6 | Western Mediterranean (Menorca channel) | 100 - 180 | Grinyó et al. 2016 |
| *Plumarella* spp. |  | 0.72 ±0.4 |  | Bering Sea | 237-356 | Miller et al. 2012 |
| *Plumarella* spp. | 5.3 | 0.46 |  | Bering Sea | 300 - 349 | Stone et al. 2006 |
| *Primnoa pacifica* | 15.3 |  |  | Gulf of Alaska | 69 - 306 | Stone et al. 2014 |
| *Primnoa resedaeformis* | 5.3 |  | 86 | Canada (Gully Canyon) | 388 - 516 | Mortensen and Buhl-Mortensen 2005 |
| *Radicipes gracilis* | 4.43 |  |  | Canada (Gully Canyon) | 404 - 535 | Mortensen and Buhl-Mortensen 2005 |
| *Swiftia pacifica* |  | 0.08 ± 0.01 |  | Bering Sea | 351 - 530 | Miller et al. 2012 |
| *Swiftia pallida* | 14 | 1.6 ± 2.8 | 17.4 | Western Mediterranean (Menorca channel) | 180 - 340 | Grinyó et al. 2016 |
| *Viminella flagellum* | 60 |  |  | Western Mediterranena (Italian Coast) | 90-200 | Angiolillo et al 2014 |
| *Viminella flagellum* |  | 0.08 ± 0.3 |  | Tirrenean Sea | 200 - 250 | Bo et al. 2013 |
| *Viminella flagellum* | 26 | 3.2 ± 5.4 | 148.3 | Western Mediterranean (Menorca channel) | 180 - 360 | Grinyó et al. 2016 |
| *Ainigmaptilon antarcticum* | 1.16 | 0.17 ± 0.31 |  | Weddell Sea | 142-363 | Orejas et al. 2002 |
| *Ainigmaptilon antarcticum* | 0.7 |  |  | Weddell Sea | 100 - 283 | Gutt et al. 2003 |
| *Ainigmaptilon sp.* | 2 | 1.2 ± 0.4 | 55.1 | Weddell Sea | 284 - 361 | This study |
| *Arntzia* sp. | 0,6 |  |  | Weddell Sea | 100 - 283 | Gutt et al. 2003 |
| *Dasystenella acanthina* | 4 | 1.8 ± 1 | 54.6 | Weddell Sea | 284 – 361 | This study |
| *Fannyella rossii* | 6 | 1.7 ± 1 | 46.5 | Weddell Sea | 284 – 361 | This study |
| *Fannyella rossii* |  |  | 21 | Ross Sea | 324 | Martinez-Dios et al. 2016 |
| *Fannyella spinosa* | 2 | 1.3 ± 0.6 | 19.3 | Weddell Sea | 284 – 361 | This study |
| Isididae |  | 0.03 |  | Ross sea | 341 - 555 | Clark and Bowden 2015 |
| Isididae | 13 | 1.8 ± 1.5 | 30.5 | Weddell Sea | 284 – 361 | This study |
| *Primnoella antarctica* | 1.5 |  |  | Weddell Sea | 100 - 283 | Gutt et al. 2003 |
| Primnoidae |  | 0.29 |  | Ross sea | 341 - 555 | Clark and Bowden 2015 |
| *Primnoisis* spp. | 4,7 |  |  | Weddell Sea | 100 - 283 | Gutt et al. 2003 |
| *Thouarella* sp. |  | 0.2 |  | Ross sea | 341 - 555 | Clark and Bowden 2015 |
| *Thouarella* sp. 1 | 10 | 1.9 ± 1.7 | 19.3 | Weddell Sea | 284 – 361 | This study |
| *Thouarella* sp. 2 | 5 | 1.5 ± 0.8 | 17.7 | Weddell Sea | 284 – 361 | This study |
| *Thouarella variabilis* | 8 | 1.7 ± 1.2 | 24.5 | Weddell Sea | 284 - 361 | This study |
| *Thouarella/Dasystenella* | 0.7 |  |  | Weddell Sea | 100 - 283 | Gutt et al. 2003 |
| Unbranched | 47 | 7.3 ± 11.5 | 111.9 | Weddell Sea | 284 – 361 | This study |

Supplementary Table S5

| Station | Latitude  (S) | Longitude  (W) | Total length (m) | Depth  (m) | Area N/S (North/South) | Data |
| --- | --- | --- | --- | --- | --- | --- |
| 170 | 74º 53.89’ | 26º 38.10’ | 1176 | 295 | N | https://doi.org/10.1594/PANGAEA.879526 |
| 136 | 75º 19.99’ | 27º 32.40’ | 1118 | 350 | N | https://doi.org/10.1594/PANGAEA.879525 |
| 128 | 75º 29.99’ | 27º 27.17’ | 1156 | 292 | N | https://doi.org/10.1594/PANGAEA.879524 |
| 49 | 76º 19.15’ | 29º 01.94’ | 679 | 251 | S | https://doi.org/10.1594/PANGAEA.879289 |
| 86 | 76º 57.41’ | 32º 59.11’ | 1310 | 284 | S | https://doi.org/10.1594/PANGAEA.879523 |
| 81 | 77º 04.82’ | 33º 39.02’ | 665 | 361 | S | https://doi.org/10.1594/PANGAEA.879522 |

Supplementary Table S6

| Morphotype | Species | Remarks |
| --- | --- | --- |
| Unbranched morphogroup:  Colonies are flagelliform; species included in this morphotype also have polyps distributed in whorls along the main stem of the colony. | *Onogorgia nodosa* (Molander, 1929) | Species allocated to several genera until Cairns and Bayer (2009) proposed the new genus *Onogorgia*^101^. This genus includes specimens with ascus body wall scales, a feature observable only under the microscope, making their identification rather difficult. |
|  | *Arntzia gracilis* (Molander, 1929) | Originally described as *Primnoella gracilis*, this species was assigned to the new Antarctic genus *Arntzia*^102^ due to significant differences with the closest genera, *Primnoella* and *Ainigmaptilon*. *Arntzia gracilis* is the only species included in the genus and it is easily recognizable. Its main features are the disposition of polyps in whorls which are fused basally, slender polyps with body sclerites aligned with the opercular ones, and, sometimes, not well-differentiated from body scales below, as they have a translucid appearance. |
|  | *Armadillogorgia* Bayer, 1980 | Two species are included in this genus *A. cyathella* Bayer, 1980 and *A. albertoi* Cerino and Lauretta, 2013. These authors conclude that specimens from this genus are not abundant; however, we cannot dismiss the possibility of having some specimens in our video footage, mainly because their general appearance can be confused with the other unbranched species. Upon closer inspection, their polyps lack opercular and marginal scales and have two perfect rows of more than forty sickle-like sclerites in the abaxial side. |
|  | *Primnoella* Gray, 1858 | Very controversial genus, for which different genera have been proposed since Bayer (1996)^103^. It differs from other unbranched species mainly in its sclerite properties such as disposition, size, ornamentations and their absence in the adaxial side, as well as the presence of non-fused polyps^104,105^. |
| Grouped bamboo corals in the Family *Isididae.* |  | Family easily recognizable by their whitish, yellow-pale axis color and conspicuous white calcareous internodes alternating with dark proteinaceous nodes. |

Supplementary Figure S1

Supplementary Figure S2


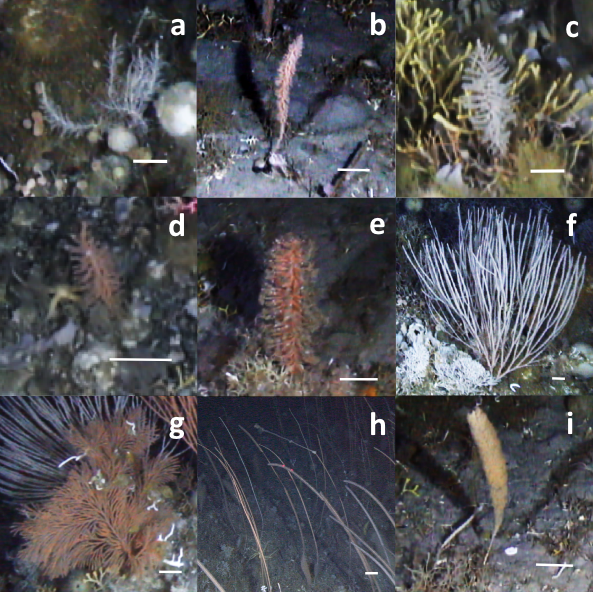


References

1. Cairns, S. D. & Bayer, F. M. A Generic Revision and Phylogenetic Analysis of the Primnoidae (Cnidaria: Octocorallia). Smithsonian Contrib Zoology **629**, 1–79 (2009).
2. López-González, P. J., Gili, J. M. & Orejas, C. A new primnoid genus (Anthozoa: Octocorallia) from the Southern Ocean. Scientia Marina **66**, 383-397 (2002).
3. Bayer, F. M. The Antarctic genus *Callozostron* and its relationship to *Primnoella* (Octocorallia: Gogonacea: Primnoidae). *P Biol Soc Wash* **109**, 150–203 (1996).
4. Cairns, S. D. The Marine Fauna of New Zealand: Primnoid octocorals (Anthozoa, Alcyonacea). Part 2. *Primnoella, Callozostron, Metafannyella, Callogorgia, Fanellia* and other genera. Biodiversity Memoir **129**, 136 p. (2016).
5. Cairns, S. D. The Marine Fauna of New Zealand: New Zealand Primnoidae (Anthozoa: Alcyonacea). Part 1. Genera *Narella*, *Narelloides*, *Metanarella*, *Calyptrophora*, and *Helicoprimnoa*. Biodiversity Memoir **126**, 71 p.
